# Clinical Features and Pathophysiology of Disorders of Arousal in Adults: A Window Into the Sleeping Brain

**DOI:** 10.3389/fneur.2019.00526

**Published:** 2019-05-17

**Authors:** Tommaso Baldini, Giuseppe Loddo, Elisa Sessagesimi, Francesco Mignani, Fabio Cirignotta, Susanna Mondini, Laura Licchetta, Francesca Bisulli, Paolo Tinuper, Federica Provini

**Affiliations:** ^1^Department of Biomedical and Neuromotor Sciences, University of Bologna, Bologna, Italy; ^2^Radiology Unit, Experimental, Diagnostic and Specialty Medicine, University Hospital S. Orsola-Malpighi, Bologna, Italy; ^3^IRCCS Istituto delle Scienze Neurologiche di Bologna, Bologna, Italy; ^4^Unit of Neurology, S.Orsola-Malpighi Hospital, University of Bologna, Bologna, Italy

**Keywords:** disorder of arousal (DoA), NREM sleep, parasomnia, pathophysiology, sleep-related behaviors, adults, video-polysomnography (VPSG)

## Abstract

**Introduction:** Disorders of Arousal (DoA) are NREM parasomnias that have been typically regarded as self-limited childhood manifestations. It is now clear that DoA can persist in adults, often presenting with distinctive characteristics. So far, few studies have described the clinical course and characteristics of DoA in adulthood, therefore a large part of their semiology is ignored. The aim of this study is to describe the clinical manifestations of DoA in an adult population and to provide a pathophysiological interpretation of their features.

**Methods:** We screened our database for all 1,600 adult (≥15 years) patients with sleep-related motor behaviors between 1995 and 2016. We identified 45 patients with typical DoA episodes, of whom a complete history, neurological examination and diagnostic video-polysomnography (VPSG) were available. All patients provided a detailed description of their episodes (with particular regards to semiology, frequency, and association with stressful life events) in different life periods. VPSG recordings were reviewed and DoA episodes were identified and assigned to three different categories according to their complexity.

**Results:** Our population was composed of 45 adult patients ranging between 15 and 76 years. Sleepwalking was reported by 86% of patients, possibly associated with complex interactions with the environment and violent behaviors in 53% of cases; distressing mental contents were reported by 64%. Recall of the episodes was reported in 77% of patients. Non-restorative sleep was reported in 46% of patients. Stress was a potential episode trigger in 80% of patients. VPSG recordings documented 334 DoA episodes. According to our classification of motor patterns, 282 episodes (84%) were Simple Arousal Movements (SAMs), 34 (10%) Rapid Arousal Movements (RAMs) and 18 (5%) Complex Arousal Movements (CAMs).

**Discussion:** Our study confirms that DoA in adulthood present with distinctive characteristics, such as non-restorative sleep, violence and complex, or bizarre behaviors. Alternative classifications of DoA based on motor patterns could be useful to characterize DoA episodes in adults, as different motor patterns often coexist in the same individual and minor episodes are more common but generally underreported by patients. Prospective studies are needed for a definitive characterization of DoA in adulthood throughout the life course.

## Introduction

Disorders of Arousal (DoA) are NREM parasomnias characterized by involuntary movements or behaviors of different complexity that occur as incomplete arousals from deep sleep (1). These events are accompanied by variable degrees of vegetative activation, automatic behaviors, misperception and reduced responsiveness to external stimuli, mental confusion and frequent retrograde amnesia (2). According to the International Classification of Sleep Disorders (ICSD-3), DoA include Confusional Arousals (CA), Sleep Terrors (ST) and Sleepwalking (SW) (1). Confusional arousals consist of confusion and disorientation without major accompanying behaviors or autonomic responses. Sleep terrors are characterized by a sudden arousal usually accompanied by a sharp scream, intense agitation and fear, confusion, and heightened autonomic discharge. Sleepwalking includes any form of complex behavior, ranging from walking to performing semi-purposeful activities. Although classified as distinct entities, DoA actually represent a spectrum of manifestations of increasing complexity (3). Indeed, these disorders share similar genetic and familial patterns, similar pathophysiology and similar priming by sleep deprivation and bio-psychosocial stressors (4).

DoA have been typically labeled as self-limited childhood manifestations that tend to disappear during adolescence (5, 6); in the last decades, however, it has progressively been understood that DoA can persist into adulthood or appear *de novo* in adults (7). A recent meta-analysis reports that the significant difference in current sleepwalking rates between children and adults is an artifact of not being observed, rather than a true effect (8). Remarkably, disorders of arousal in adults exhibit different characteristics from childhood DoA (9). They are more often associated with excessive daily sleepiness (with an impact on daytime activities) and violent or potentially harmful behaviors, which are rare in childhood DoA (9–13). These can include running into walls and furniture, trying to escape imaginary threats, leaving one's house, destruction of property, driving motor vehicles, suspected suicide, and even homicide or attempted homicide (14–16).

Additionally, the semiological manifestations of adult DoA include a spectrum of motor patterns of different complexity and duration that are not contemplated in the ICSD-3 classification, so that their video-polysomnographic (VPSG) recognition and interpretation can sometimes be problematical. Indeed, while in typical cases the diagnosis of DoA can be done with clinical history alone, in adult DoA diagnostic uncertainty often exists and VPSG is needed (7, 17). This is particularly true in the differential diagnosis with Sleep-related Hypermotor Epilepsy (SHE), a form of focal epilepsy in which motor seizures appear predominantly during sleep (7, 18–21). Therefore, knowledge of the precise characteristics of DoA in adults is essential for a correct diagnosis.

So far, few studies have described the clinical course of DoA in adulthood and have been usually performed after medical or psychological therapy (10, 22, 23). Moreover, few studies have reported specific description of DoA episodes in adulthood, therefore a big part of their semiology remains neglected, with negative repercussions to the diagnosis of these conditions. (2, 10, 13, 20, 24) The aim of this study is to describe the clinical features of DoA in an adult population—with particular attention to their trend during lifetime, the semiology of the episodes and the presence of predisposing and precipitating factors. Additionally, we provide a pathophysiological interpretation of these clinical characteristics on the basis of the most recent evidence from the literature.

## Materials and Methods

### Patient Selection and Interview

We screened our electronic database for all 1,600 patients ≥15 years of age who underwent video-polysomnography (VPSG) at our sleep laboratory for complex motor behaviors during sleep in the time period from 1995 to 2016. We identified all patients presenting with at least one typical DoA episode according to the ICSD-3 criteria. We excluded those patients for whom a complete personal, family and medical history and a neurological examination were not available. All patients underwent a telephone interview and subsequently a semi-structured face-to-face interview (along with any potential witness of the events), paying particular attention to the description of DoA episodes. Patients were also asked to report the frequency of DoA episodes in different periods of their life (5–15 years; 15–25 years; 25–35 years; 35–50 years). For each life period, patients were subdivided into two subgroups: a high-frequency group (more than one DoA episode per week) and a low-frequency group (<1 DoA episode per week). For each of these periods we also explored the presence of stressful life events.

### VPSG Analysis and Classification of Motor Patterns

We reviewed the VPSG recordings of all patients and identified any DoA episodes. We classified the episodes on the basis of their main motor pattern according to Loddo et al. dividing them into three groups with increasing intensity and complexity: pattern I or simple arousal movements (SAMs), pattern II or rising arousal movements (RAMs) and pattern III or complex arousal with ambulatory movements (CAMs), i.e., sleepwalking. SAMs are further subclassified in A: head flexion/extension; B: head flexion/extension and limb movement; C: head flexion/extension and partial trunk flexion/extension (25). Sleep stages were scored according to the standard American Academy of Sleep Medicine (AASM) criteria, and the percentages of NREM and REM sleep stages and sleep efficiency (the percentage of total sleep time/time in bed) were evaluated.

### Statistical Analysis

Continuous variables were presented as mean ± standard deviation (SD) and categorical variables as absolute frequency and relative frequency (%).

## Results

### Clinical Data

Our population included 45 DoA patients ≥ 15 years (25 males and 20 females). At the time of VPSG, patients' ages ranged from 15 to 76 years (mean ± SD: 33±17 years); only one patient was younger than 18. DoA onset was at 13 ± 11 years (range 5–58). We didn't find any differences between males and females in our cohort of patients.

On the base of the age of onset, it was possible to distinguish between two set of patients: relapsing and adult-onset DoA. Forty patients (89%) have had a disease onset before 18 years of age but persisting over time (i.e., relapsing DoA), with a mean duration of disease of 21 ± 14 years (range: 1–64). At the time of our observation, those patients had a mean age of 31 ± 16 (range 13–74). The remaining five patients (11%) have had a disease onset after 18 years of age (i.e., adult-onset DoA), mean 36 ± 15; range: 19–58; no one after 65. At the time of our observation, those patients had a mean age of 40 ± 14 (range: 24–76), with a mean duration of disease of 10 ± 11 years (range: 2–20). There were no differences between the two groups regarding the number of episodes per night or the VPSG data. Unfortunately, due to the small number of the cohort, it wasn't possible to statistically compare the two groups.

### Family and Personal History of the Patients

Twenty patients (44%) reported one or more first-, second- or third-degree relatives positive for DoA. DoA in relatives were usually described as sporadic, occurring mostly in childhood and persisting into adulthood only in two cases. Six patients reported a family history for sleep terrors (13%), ten for sleepwalking (22%) and four for confusional arousal (8%). One patient had a positive family history for epilepsy. All patients reported normal birth and psychomotor development. Two patients reported infantile febrile convulsions; one patient underwent surgical removal of a pilocytic astrocytoma of the IV ventricle and posterior brainstem 4 years after DoA onset. Eight patients (16%) reported headache. Nineteen (42%) patients reported concomitant medical diseases, 8 (16%) reported headache and twelve (26%) had been suffering from psychiatric disorders. Seven patients (15%) reported other concomitant sleep disorders (Table 1).

**Table 1:** Clinical features of the studied cohort.

**Concomitant medical disease**	**Number of patients (%)**
Allergic diseases	6 (13%)
Thyroid diseases	5 (11%)
Gastritis	3 (6%)
Hypertension	3 (6%)
Previous cancer	2 (4%)
**CONCOMITANT NEUROLOGIC DISEASE**	
Migraine	4 (8%)
Tension-type headache	4 (8%)
**CONCOMITANT PSYCHIATRIC DISEASES**	
Depression	5 (11%)
Anxiety	5 (11%)
Post-traumatic stress disorder	2 (4%)
**CONCOMITANT SLEEP DISORDERS**	
Insomnia	3 (6%)
Bruxism	2 (4%)
Obstructive sleep apnea (AHI 5-10)	2 (4%)

*AHI, apnea-hypopnea index*.

### Clinical Description of DoA Episodes as Reported by the Patients

The type of motor activation and of interaction with the environment and the presence of mental activity during the episode in our cohort are summarized in Table 2.

**Table 2:** Clinical characteristics of DoA episodes reported by patients.

**Motor activity**	**Number of patients (%)**
Sleepwalking	39 (86%)
Leaving the room	15 (33%)
Leaving the house	6 (13%)
Sitting in the bed	39 (86%)
**BEHAVIORS AND DEGREE OF INTERACTION**	
Sleep talking	44 (97%)
Interaction with the environment	31 (68%)
Simple actions mimicking daily activities	20 (44%)
Bizarre behaviors	23 (51%)
Violent behaviors	24 (53%)
Interaction with other people	29 (64%)
Verbal interaction	17 (33%)
Physical interaction	12 (26%)
**MENTAL ACTIVITY**	
Frightening or distressing content	29 (64%)
Quite mental activity	6 (13%)
Recall for the episode	35 (77%)

#### Motor Activity

Thirty-nine patients (86%) reported they could get out of bed and wander around during a typical DoA episode. Fifteen patients (33%) could leave their room and six of them left their house at least once. Thirty-nine (86%) reported also episodes during which they could stay in bed, sit up and often speak or shout, look around, turn on the light or handle objects. For twenty patients (44%) the bed partner described a frightened facial expression during DoA episodes.

#### Behaviors and Interaction With the Environment

Thirty-one patients (68%) described some kind of interaction with the environment, with variable degrees of complexity. Twenty patients (44%) exhibited simple actions or behaviors mimicking daily activities such as: turning on the light, looking at or using the mobile phone, opening a door or looking out of the window, raising the blinds, checking if the door was locked, ringing the bell or knocking on the door of a neighbor, getting dressed, preparing breakfast, laying the table, taking actions related to working activity, washing clothes, preparing luggage, doing their makeup, going up or down the stairs, climbing on the bike in the garage. Twenty-three patients (51%) reported also more bizarre behaviors: hiding objects (e.g., a watch, a remote control), tinkering with components of the furniture in the room (e.g., handling, unplugging or lifting and then letting a lamp fall to the floor, moving a wardrobe or the bed, pulling down a shelf, lifting a heavy compact disc holder off the ground, overturning a bedside table, trying to take the door off its hinges, looking for something—a fox—inside the wardrobe, punching the door or a shutter), cutting something with a knife or scissors (an electric cable, the sheet), removing the sheets from the bed and piling them on the ground, wrapping themselves in the sheets, throwing a pillow, climbing a spiral staircase, climbing over a window, waving their arms and knocking them on the bed or on the ground, pushing hands and feet against the wall, getting on a couch, acting as if throwing something (such as some water) toward a lamp.

Twenty-four patients (53%) reported violent and/or injuring behaviors, including simple dream enactment and/or complex behaviors potentially or actually dangerous for the patient and/or bed partner. Examples include hitting or tripping over objects in their room (the door, a glass, the dog, the dresser) and in some cases falling on the ground, falling out of bed, or trying to climb over a window. Injuries included cracked ribs in three cases, knee dislocation in one patient, a cut to the chin requiring stitches in one patient, and more frequently other minor lesions (excoriation, soreness in the bruised area for a few days after the trauma).

All patients except one (97%) reported they could utter vocal sounds, including moaning, mumbling, shouting, saying a few words (more or less intelligible), articulating complex sentences or even singing. Twenty-nine patients (64%) reported that during DoA episodes they could interact with their bed partner or other family members, being verbal in 33% of subjects; this included speaking with the bystanders (often excitedly) either using a few words or more complex and articulated sentences. Physical interaction was reported by 26% of subjects and mostly described as aggressive or even violent.

#### End of the Episodes

Awakening from episodes was possible (although not constant) in twenty-three patients (51%) and could be caused both by internal or external stimuli, such as shouting or feeling pain after a fall. Once awake the majority of patients reported they felt confused and disorientated.

Twenty-one patients (46%) complained of non-restorative sleep and twenty-two (48%) reported that they often felt tired during the day.

#### Mental Activity Associated With DoA Episodes

Thirty-five patients (77%) reported that, even if not constantly, they could recall some kind of mental activity at the end of the episode. Of them, twenty-nine (64%) reported frightening and distressing contents, variably alternating with neutral contents in four of them. Fearful contents included someone chasing or trying to kill the patient, the ceiling falling on the patient, a truck running over the patient, mice infesting the house, being inside a box from which it was impossible to escape, a fire, walls crashing during an earthquake, thieves entering the house or a fox in the room. Because of these mentations, some patients could rise abruptly, jump out of bed, run and/or carry out protective behaviors like kicking or moving the arms. Finally, six patients (13%) reported a quiet mental activity (such as reliving the experiences of the same day or being late for work with colleagues waiting outside in the street) in association with DoA episodes.

### DoA Frequency and Lifetime Occurrence

Almost all patients reported that DoA episodes usually occurred in the first or in the central part of the night. All patients reported that DoA frequency and intensity varied during the course of the disease, alternating periods of DoA high frequency (nightly or weekly episodes) with free periods lasting from few weeks to years. Four patients reported a free period more than 5 years long.

At the time of our VPSG, 18 patients (40%) reported nightly episodes; 17 patients (38%) had more than one episode per week; seven patients (15%) <1 episode per week but more than one per month; 3 patients (7%) reported <1 episode per month.

The detailed patient distribution according to DoA episode frequency in the different life periods is reported in Table 3.

**Table 3:** Patient distribution according to the different DoA episodes frequency in the different life periods.

**Life period**	**Number of patients (%) with a high frequency**	**M**	**F**	**Number of patients (%) with a low frequency**	**M**	**F**
5–15 years	10 (30%)	6	4	23 (70%)	13	10
15–25 years	17 (51%)	8	9	16 (49%)	6	10
25–35 years	13 (50%)	7	6	13 (50%)	7	6
35–50 years	2 (20%)	1	1	8 (80%)	5	3

M, males; F, females.

High frequency is defined as ≥ 1 episode/week, while low frequency < 1 episode/week.

*The total number of patients in the different life periods can be <45 due to the different age of onset and the different degree of episodes' recall*.

### Triggering Events and Predisposing Conditions

Two patients (4%) reported a head trauma before the onset of DoA. Thirty-six patients (80%) reported that both stressful and positive emotional events or situations could increase the frequency of DoA episodes, whereas sleeping in familiar places or with a light on could reduce it.

### VPSG Analysis: Sleep Parameters and Motor Patterns

Our cohort of 45 patients underwent a total of 103 VPSG showing 334 DoA episodes. Of these, 72% appeared in the first third of the night and 80% emerged from slow-wave sleep. In all episodes, we didn't identify any features suggestive of epilepsy, such as asymmetric o dystonic posturing, kicking, cycling and rocking body movements, which are required to make a diagnosis of SHE (19).

**Figure 1:**
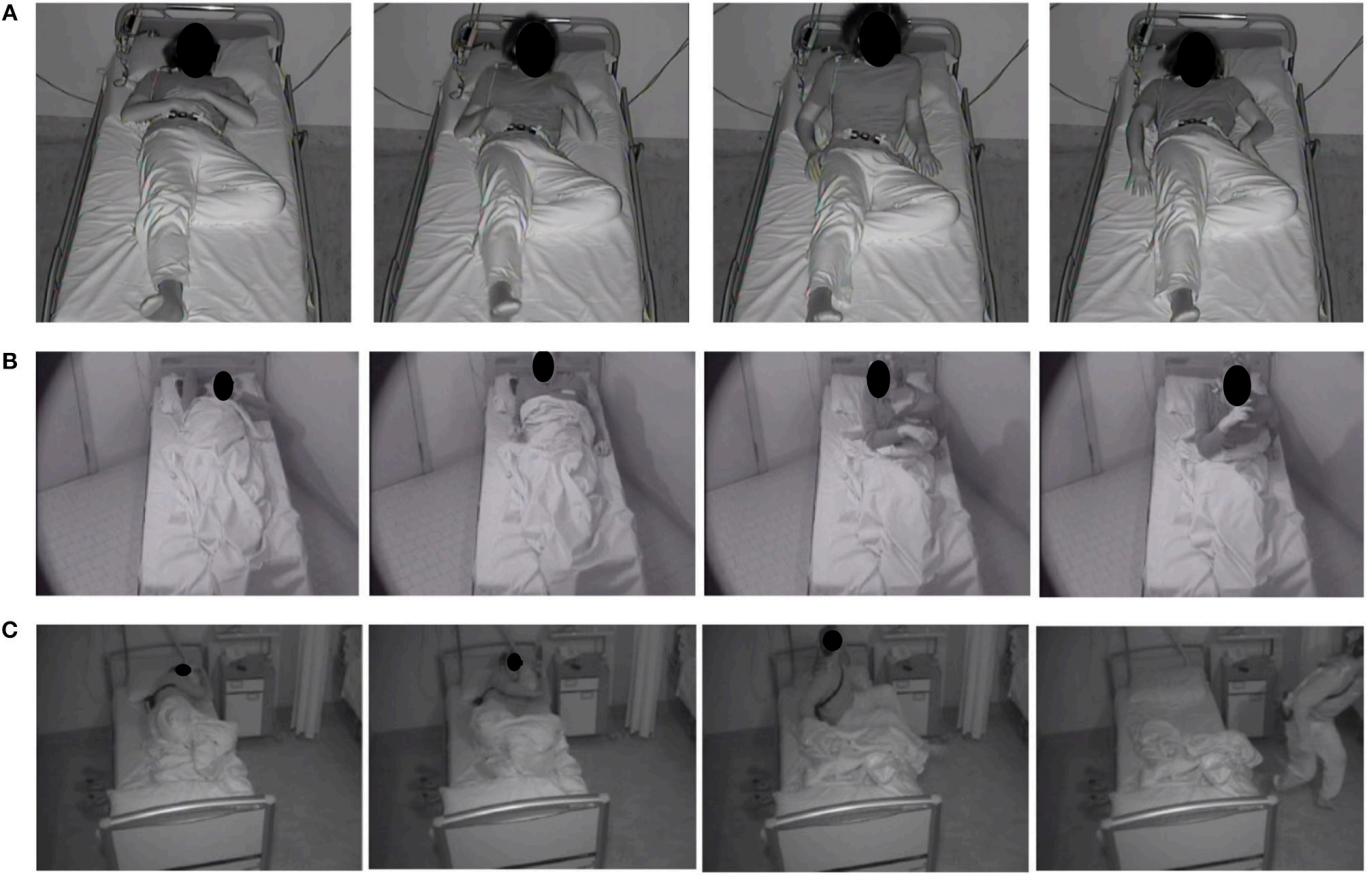
Photographic sequences of the three different motor patterns of DoA in adults. **(A)** Simple Arousal Movement (SAM): this pattern was characterized by head flexion and/or head extension and/or limb movement; **(B)** Rising Arousal Movement (RAM): the core feature of this pattern was a trunk flexion followed by sitting with feet in or out of the bed; **(C)** Complex Arousal with ambulatory Movements (CAM): this pattern was characterized by sitting up, getting out of bed, and walking.

Sleep efficiency, light and deep non-rapid eye movement (NREM) and rapid eye movement (REM) sleep stages, and REM latency were normal in all patients (Table 4). In the two patients with OSAS the episodes of DoA didn't occur after an obstructive apnea. According to Loddo's classification of motor patterns, 282 episodes (84%) were SAMs (of which 60 SAM-A, 128 SAM-B, and 94 SAM-C), 34 (10%) RAMs and 18 (5%) CAMs (Figure 1) (25).

**Table 4:** Sleep parameters in our DoA patients cohort.

**Sleep parameters**	**Values**
Total sleep time, minutes	411 ± 103
Sleep efficiency, % (NV > 85)	86 ± 11
REM latency, min (NV 60–120)	102 ± 56
Sleep stage 1, % (NV 2- 5)	8 ± 6
Sleep stage 2, % (NV 45–55)	45 ± 10
Sleep stage 3, % (NV 15–25)	25 ± 10
Sleep stage REM, % (NV 20–25)	22 ± 7
PLMI	2 ± 6

*NV, normal value; REM, rapid eye movement; PLMI, periodic limb movement index*.

## Discussion

### Clinical Characteristics of DoA in Adults

In our cohort of patients DoA began at an average of 13 years and persisted at the time of our VPSG (to a maximum of 76 years), supporting the hypothesis that DoA could represent a lifelong sleep disorder, arising in childhood and/or adolescence and persisting in adulthood at a variable level of intensity. The frequency and intensity of the episodes varied greatly during lifetime and among different patients. Quite often DoA showed a fluctuating course, alternating periods of high frequency and intensity of the episodes followed by intervals of months or even years free from episodes. Such fluctuations may partially explain the long delay between episodes' onset and the diagnosis.

The accurate description of DoA episodes provided by the patients or by their witness revealed a considerable degree of variability and complexity that are not usually observed during laboratory VPSGs (26–28). This supports the need for an extensive history taking, both from the patients and from any bed partner, in order to make a correct diagnosis and also to identify and possibly prevent any dangerous behaviors during an episode of DoA.

The ICSD-3 categorizes DoA as confusional arousals, sleep terrors and sleepwalking (1). However, as underlined by our study, the semiological manifestations of DoA in adults comprehend a spectrum of motor patterns of different complexity that often coexist in the same individual. Therefore, it can be difficult to make a definite diagnosis of a DoA subtype in adults. Instead, semiological-based classifications could be more precise in the description of the episodes. Loddo et al. recently proposed a classification of the motor patterns of DoA in adult patients, identifying three categories with increasing complexity: pattern I or SAMs, pattern II or RAMs and pattern III or CAMs (25, 29). The majority of our patients reported to suffer mainly from complex behaviors (sleepwalking and structured and bizarre actions), however during VPSGs the number of minor episodes (SAMs) largely exceeds that of the major episodes (RAMs and CAMs). This can be explained with the following considerations. First, patients probably do not recall the majority of the minor episodes, as they are too short and devoid of mental activity to be remembered after awakening; therefore, SAMs are typically underreported by patients. Secondly, patients usually exhibit more than one motor pattern, either in different periods of the night or in different life periods. It is therefore expected that the majority of VPSG recordings of adults complaining of sleepwalking will document only minor episodes. Also, it is possible that in a consistent number of adults who have a positive past medical history for DoA in childhood, such “minor” episodes are not recognized but actually persist at a subclinical level. This is reasonable as the mechanisms responsible for such conditions and therefore the predisposition to suffer from DoA are life-lasting (see below). Hence, we believe that the proposal of a classification based on motor patterns could be useful in the diagnosis of DoA in adults. For example, in the setting of a patient whose history is suggestive of sleepwalking but without documentation of major episodes, recording of SAMs could corroborate the diagnosis of a DoA. Still, further studies are necessary to validate the usefulness of this model in clinical practice.

Violent behaviors, posing threats to the patient itself or to others, were reported in more than half of our cases. These data confirm that DoA in adults can be a major cause of injuries during sleep, complicating the differential diagnosis with other motor behavioral manifestations during sleep (9). However, the high variability of the episodes' semiology, intensity and frequency over time are typical of DoA and therefore represent a critical diagnostic feature to differentiate DoA from epilepsy. On the other hand, a progressive increase of the episodes' frequency and the presence of motor stereotypy typically suggest Sleep-related Hypermotor Epilepsy (SHE) (20, 21, 30–33).

Once awake, the majority of patients reported that they felt confused and disorientated, and more than half of patients could not recollect the episode. Approximately one-third had complete amnesia. This raises fundamental questions about the medico-legal and forensic implications of DoA, given the neurophysiologic and cognitive states that characterize patients during such episodes (34).

When recollected, however, more than half of the patients reported distressing mental contents during the episodes. This is consistent with the view that in adults at least some, if not all, the episodes of DoA may originate from cortical activity and be associated with NREM sleep mentation (see below).

Finally, almost half of the patients complained of non-restorative sleep and daytime tiredness, opening the question if non-refreshing sleep is the only reason explaining the presence of daytime tiredness or if it is an intrinsic characteristic of the disorder itself (35).

### Pathophysiology of DoA in Adults

Disorders of arousal result from a NREM sleep-wake state dissociation (36). In the last decades it has progressively become clear that wake and sleep are not mutually exclusive, and admixture of features of the different states can occur (37, 38). During transitions between NREM sleep and wakefulness (as it occurs during arousals) a temporary, pathological dissociation of states can occur across different brain structures, resulting in a state of altered consciousness manifesting as DoA (39).

**Figure 2:**
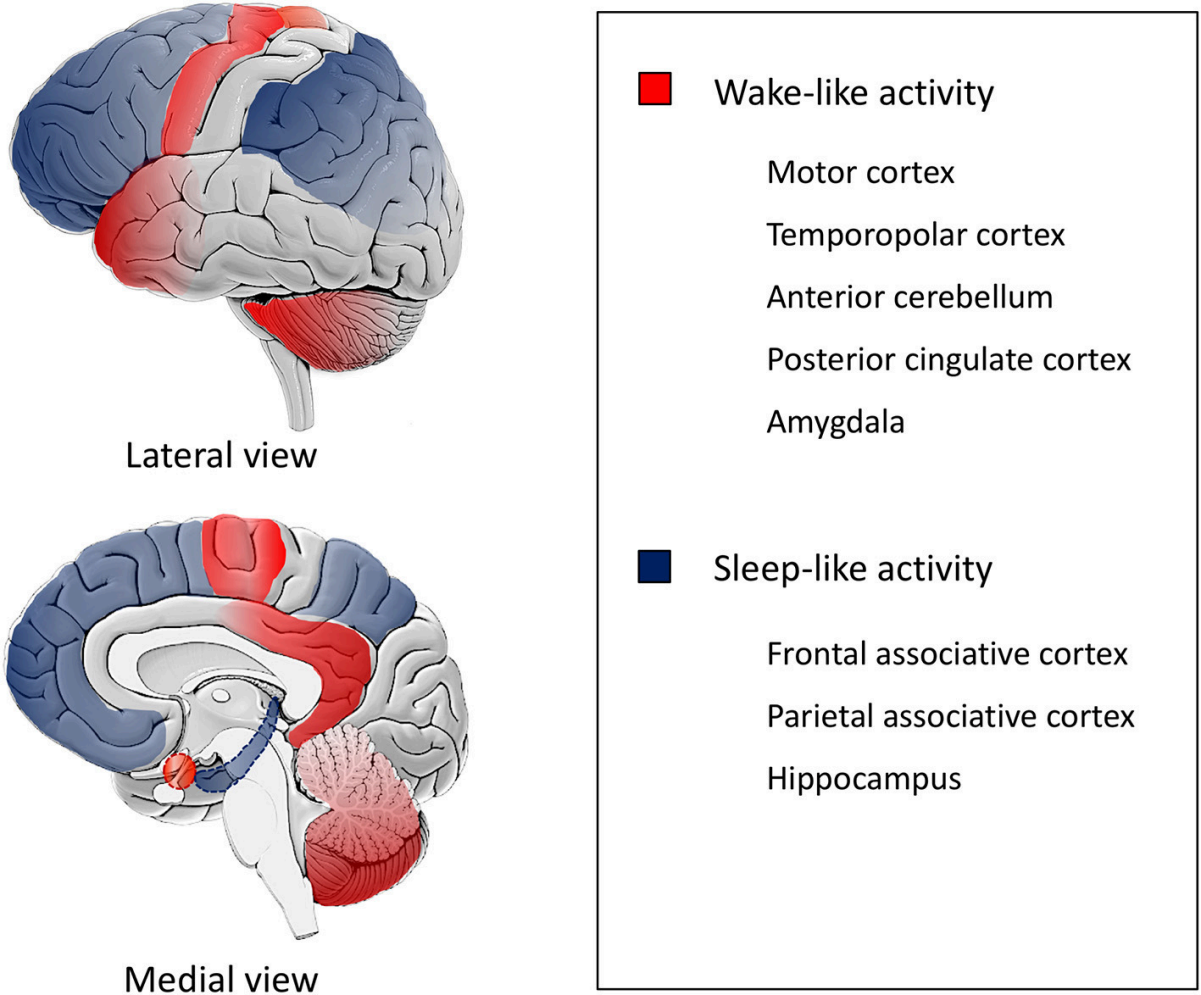
Schematic representation of state dissociation during DoA. The figure is a schematic representation based on data from SPECT and stereo-EEG studies, (41–43) illustrating state dissociation in disorders of arousal (DoA), i.e., co-occurrence of different local activity patterns in the human brain. Motor, temporopolar, anterior cerebellar, posterior cingulate cortices and the amygdala exhibit a wake-like activity (red) while fronto-parietal associative and hippocampal cortices show a sleep-like activity (blue).

As depicted by the description of the episodes and specifically the most bizarre ones, patients appear to be simultaneously awake (with retention of their motor and behavioral functions) and asleep (with impairment of cognition, judgment and memory for the events) (40). Indeed, a SPECT study performed during sleepwalking showed a decrease in regional blood flow in the fronto-parietal associative cortices, and an increase in blood flow in the posterior cingulate cortex and in the anterior cerebellum (41). Also, stereo-EEG studies during DoA identified local fast wake-like EEG activity on the motor, cingulate, insular, temporopolar and amygdalar cortices and sleep-like EEG with increased delta activity on the fronto-parietal associative cortices and in the hippocampus (Figure 2) (42, 43).

Patients with DoA seem to be predisposed to state dissociation, probably due to abnormal neuronal excitability in different cortical areas (44, 45). A high-density EEG study and a SPECT study showed persistent, localized changes in neuronal excitability of DoA patients: specifically, an increased arousability of motor and limbic areas, in contrast with a reduced arousability of associative cortices (especially frontal) (40, 45–47). The impaired inhibitory control of motor systems and increased motor impulsivity could partly explain the violent behaviors observed in DoA patients (47, 48).

Hence, during arousals from SWS, motor and limbic areas are more easily aroused while associative areas more difficulty transition into wakefulness, especially in conditions of increased SWS pressure.

Therefore, two types of conditions are thought to increase the likelihood of DoA episodes in predisposed individuals. First, all conditions increasing the amount of SWS, such as sleep deprivation, physical or emotional stress, fever and medications affecting sleep increase the likelihood of DoA episodes occurring. Indeed, most of our patients reported that the episodes occurred mainly in the first half of the night, which is consistent with a physiological higher proportion of slow wave sleep (SWS) during this period. Secondly, arousal by whatever mechanism (internal or external) can precipitate a DoA episode (1). The list includes comorbid sleep disorders (particularly Obstructive Sleep Apnea Syndrome, OSAS, and Periodic Limb Movement Disorder, PLMD), bladder distention, physiological ending of a sleep cycle, mental activity and noises (49). In our study, we found a significant relation between stress and DoA episode frequency, as one would expect from the effects of stress on sleep, i.e., SWS reduction (and therefore increase in SWS pressure) and an increase in the number of arousals (50).

Other physiologic phenomena have been proposed to contribute to disorders of arousal (1). Indeed, some behaviors observed in DoA resemble stereotyped, archaic behaviors (such as defensive postures, violent gestures and feeding) that result from the activation of neural circuits (mainly subcortical), namely central pattern generators (CPGs). As these structures in humans are largely under neocortical control, it has been suggested that some manifestations of DoA could result from CPG disinhibition, as a result of prefrontal cortex dysfunction (51, 52). In many cases of adult DoA, however, more complex and learned behaviors, of likely cortical origin, are performed. Interestingly, these can be accompanied by a congruent dream-like mentation (34). Our study confirmed these data, as the majority of patients exhibited quite intricate behaviors (e.g., getting dressed, preparing breakfast, doing their makeup) and were able to recall mental contents (often distressing) related to the episodes. These data suggest a role of NREM sleep dreaming in the genesis of some episodes of DoA, which could then represent “dream-enactment behaviors” (34, 53–55).

In conclusion, DoA occurs when internal or external precipitating factors trigger the episodes in predisposed individuals. The precise cause of the “pathological” state dissociation and cortical abnormal excitability, however, is unknown, but probably results from an interplay of genetic and environmental factors (56). A familial predisposition have been reported suggesting a genetic basis for DoA (23, 57, 58) and family studies have recognized several potential candidate genes, including the adenosine deaminase gene (59).

## Conclusion

In this study we described the clinical and pathophysiological characteristics of DoA in adults, underlying their unique characteristics such as violent behaviors and non-restorative sleep. Our results seem to suggest that DoA could be lifelong disorders, whose frequency oscillates over time, often in association with stress.

Our study was performed in the absence of any treatment and included data of patients during different periods of life and reported specific description of DoA episodes. However, there are some limitations to the present study: particularly, the small sample of the cohort and the retrospective self-evaluation of frequency and severity of the episodes, possibly not reflecting the real spectrum of DoA manifestations. The episodes are followed by variable degrees of amnesia, therefore the information may not be entirely accurate. We tried to minimize this risk by requiring a face-to-face interview with a witness as an inclusion criterion. Also, patients attending a sleep center are often not really representative of the general population because they could have more frequent and/or severe episodes. Therefore, prospective and longer follow-up studies are needed for a definitive characterization of clinical course of DoA in adulthood.

We encourage the implementation of the classification of motor patterns of DoA as they could help in the diagnosis of these conditions, since different patterns often coexist in the same patients and episodes of minor intensity are generally underreported by patients.

## Ethics Statement

This study was carried out in accordance with the recommendations of the International Good Clinical Practice guidelines, ethical committee Area Vasta Emilia Centro (CE-AVEC), with written informed consent from all subjects. All subjects gave written informed consent in accordance with the Declaration of Helsinki. The protocol was approved by the CE-AVEC (protocol number 17176, 14/12/2017).

## Author Contributions

TB: data collection, literature revision and critical revision of the manuscript. GL and ES: data collection, writing of the manuscript. FM: VPSG analysis. FC, SM, LL, FB, and PT: data collection and interpretation of results. FP: data collection, literature revision, and revision of the manuscript.

### Conflict of Interest Statement

GL received honoraria as sub-investigator activities from UCB Pharma. FB received honoraria from EISAI for speaking engagements and consultancy. PT received honoraria from UCB, EISAI, Liva-Nova for speaking engagements and consultancy. FP received honoraria from Bial, Sanofi, Vanda Pharmaceutical, Fidia, Zambon, Eisai Japan, Italfarmaco for speaking engagements and consultancy. The remaining authors declare that the research was conducted in the absence of any commercial or financial relationships that could be construed as a potential conflict of interest.
